# A cadaveric and sonographic study of the morphology of the tibialis anterior tendon – a proposal for a new classification

**DOI:** 10.1186/s13047-019-0319-0

**Published:** 2019-02-01

**Authors:** Łukasz Olewnik, Michał Podgórski, Michał Polguj, Mirosław Topol

**Affiliations:** 1 Department of Normal and Clinical Anatomy, Interfaculty Chair of Anatomy and Histology, Medical University of Lodz, Lodz, Poland; 2 Department of Diagnostic Imaging Lodz, Polish Mother’s Memorial Hospital Research Institute, Lodz, Poland; 3 Department of Angiology, Interfaculty Chair of Anatomy and Histology, Medical University of Lodz, Lodz, Poland

**Keywords:** Anatomical study, Cadaveric study, New classification, Radiological study, Tibialis anterior tendon, Tibialis anterior muscle, Ultrasound

## Abstract

**Background:**

The tibialis anterior tendon (TAT) presents little morphological variation. The tibialis anterior muscle originates at the lateral condyle of the tibia, the proximal one-third to two-thirds of the lateral surface of the tibia shaft, and the anterior surface of the interosseous membrane and inserts to the medial cuneiform bone and first metatarsal. The aim of our work is to classify types of TAT insertion by two complimentary methods - anatomical dissection and ultrasound examination.

**Methods:**

In the first part, classical anatomical dissection was performed on 100 lower limbs (50 right, 50 left) fixed in 10% formalin solution. The morphology of the insertion of the tendon was evaluated and the muscle was subjected to the appropriate morphometric measurements. In the second part, the morphology of the TAT insertion was evaluated in 50 volunteers with ultrasound.

**Results:**

The tibialis anterior muscle was present in all specimens. In the cadavers, five types of insertion were observed, the most common being Type V: a single band attaching to the medial cuneiform bone (32%). In the sonographic part, Type IV was not observed; however, an additional insertion type was recognised (Type VI), which was characterized by two identical bands attached only to the medial cuneiform bone. The most common type identified by ultrasound was Type II (35%).

**Conclusion:**

The tibialis anterior tendon presents high morphological variability that can be observed both in cadavers and in vivo by ultrasound examination.

**Level of evidence:**

II Prospective Comparative Study

**Electronic supplementary material:**

The online version of this article (10.1186/s13047-019-0319-0) contains supplementary material, which is available to authorized users.

## Introduction

The tibialis anterior muscle (TAM) has a prismatic belly, originates at the lateral condyle of the tibia, proximal one-third to two-third of the lateral surface of the tibia shaft, and on the anterior surface of the interosseous membrane [1]. The muscle belly becomes the tibialis anterior tendon (TAT), which inserts into the medial cuneiform bone and the first metatarsal bone [1]. The TAM provides the strongest dorsiflexion among muscles from the anterior compartment of the leg, with its main function being the dorsal flexion of the ankle and supination of the foot. Such dorsiflexion is essential to gait, as this movement clears the foot off the ground during the swing phase [2].

Recent years have seen a growth in interest in physical activity by non-professional athletes, and traumatic/atraumatic ruptures and tendinopathies of the tendons are quite commonplace [3–5]*.* Usually, in cases of a complete tendon rupture, an accurate diagnosis can be made solely on the basis of a palpation examination; however, before any procedure can be performed, these findings have to be confirmed with some form of imaging technique. Moreover, palpation is insufficient for diagnosing tendinopathy, partial tears or bursitis, therefore, diagnostic imaging (USG with Color Doppler, MRI, or CT-scanners) seems to be necessary [3].

Ultrasonography (US) is an effective technique for evaluating soft tissues that are localised superficially and are not obscured by gases or bones. US, therefore, is ideal for obtaining high resolution evaluations of the TAT along its entire course. It is a low-cost technique that is easily accessible, has no contraindications for testing, and can also be used for evaluation of muscle contractility and tendon traction [6].

There are two treatment options for traumatic tendon rupture: conservative treatment with foot orthoses, and surgical treatment with TAT reconstruction [6, 7]. Reconstruction of the TAT used to restore ankle dorsiflexion and inversion includes end-to-end repair, tendon transfer, or allograft augmentation [6–9]. In order to restore the natural lever function of the tibialis muscle, it is necessary to return the tendon to its correct anatomical position [10–12]. In contrast to tears, first-line intervention in tendinopathy of TAT consists of rehabilitation. The most commonly-used approach for lower limb tendinopathies combines eccentric training with manual therapy, kinesiology taping, isometric and stretching exercises, electrotherapy or improvement of lumbo-pelvic control [13–16]. However, all these methods require detailed anatomical knowledge on the course and insertion of the tendon. The choice of rehabilitation procedures needs to consider the location of insertion (e.g. in cases of enthesopathy) and the course and force vector of the TAT. Although classifications of TAT morphology do currently exist [10, 12, 17–19], they are inconsistent and lack precision with regard to the site of distal insertion.

The aim of our work was to classify types of TAT insertion by two complimentary approaches: anatomical dissection of cadavers and ultrasound examination of healthy volunteers. Knowledge of the variability of the attachment of the TAT and the ability to evaluate it with ultrasound may be used to complement current methods of patient management.

## Material and methods

The study was divided into two parts: anatomic and sonographic. The anatomical study procedure was approved by the Medical University of Lodz Bioethical Commission (agreement no. RNN/297/17/KE), and the ultrasound study procedure was approved by the Polish Mother’s Memorial Hospital Research Institute Bioethical Commission (agreement no. 54/2018).

### Anatomic study

One hundred lower limbs (50 paired, 62 male, 38 female) were obtained from adult Caucasian cadavers, and fixed in 10% formalin solution before examination. The mean age “at death” of the cadavers was 63.8 years (35–88). The cadavers were the property of the Department of Normal and Clinical Anatomy of the Medical University of Lodz, following donation to the university anatomy program. The inclusion criteria comprised sufficient specimen quality and the lack of evidence of surgical intervention in the examined area; which was needed to allow for complete identification of the tendon insertion. Limbs with hallux valgus were excluded from the analysis. A dissection of the leg and foot area was performed by traditional technique [20–23].

Dissection started from the area of the leg, with the removal of the skin and superficial fascia to the crural fascia. Following this, the skin and subcutaneous tissue of the foot were removed, and then, starting proximal to the retinaculum, as much of the crural fascia as possible was removed without tearing the muscle bellies. The bellies and muscle tendons were then cleaned from the medial to lateral side. The tendon was very precisely dissected to the bone attachment itself. The course of each tendon was checked very carefully.

Upon dissection, the morphological features of the TAM were assessed:

- The types of TAT insertion.
- Morphometric measurements of the TAM.

An electronic digital caliper was used for all measurements (Mitutoyo Corporation, Kawasaki-shi, Kanagawa, Japan). Each measurement was carried out twice with an accuracy of up to 0.1 mm and the mean value of the two measurements was used in further analyses. The size of each band was measured. If they differed by more than 20%, one was recognised as smaller and the other as larger. This assumption was made to improve the functional value of the classification. In almost all cases, one band was found to be larger than the other; however, such small differences do not always have a significant effect from the mechanical point of view. A limit of 20% was chosen because, in our experience, such a difference can be identified by visual inspection alone, thus allowing the classification to be used in further works where surgeons might not have the time or equipment required for more precise measurement.

### Sonographic study

Fifty healthy volunteers (23 women, 27 men) were invited to receive an ultrasound evaluation of the TAM and TAT. The mean age of volunteers was 39 years (25–55). Exclusion criteria included any injury to the TAM/TAT which might interfere with their morphology such as foot and ankle deformities (particularly halux valgus) or skin wounds/lesions that enabled application of sonographic gel. The patients were informed about the details of the tests, as well as the possibility of leaving the study at any time without giving a reason. All volunteers gave signed written permission to perform the tests.

Both feet were examined using a Samsung RS80 Expert apparatus with a 16 MHz linear probe. The patients were placed in a supine position with flexed hip and knee joints, and muscle was scanned through the belly and the tendon. The area of the tendon was assessed by measuring a cross-section of the main tendon and its accessory bands at their origin. The localisation of the distal attachment was noted. Each measurement was carried out twice with an accuracy of 0.1 mm. The mean value of these two measurements was used in further analyses.

#### Statistical analysis

The statistical analysis was performed using Statistica 12 software (StatSoft Polska, Cracow, Poland).

The normality of the continuous data distribution was checked with the Shapiro-Wilk test. The difference between the five TAT classifications were assessed using caliper vitro morphology measurements. The differences between mean tendon length, width and thickness were analysed using a Kruskal-Wallis test by ranks with dedicated post hoc test was used to compare these measurements between each of the TAT types (data was not normally distributed).

A *p*-value lower than 0.05 was considered significant. The results are presented as mean and standard deviation unless otherwise stated.

## Results

### Anatomic study

**Fig. 1:**
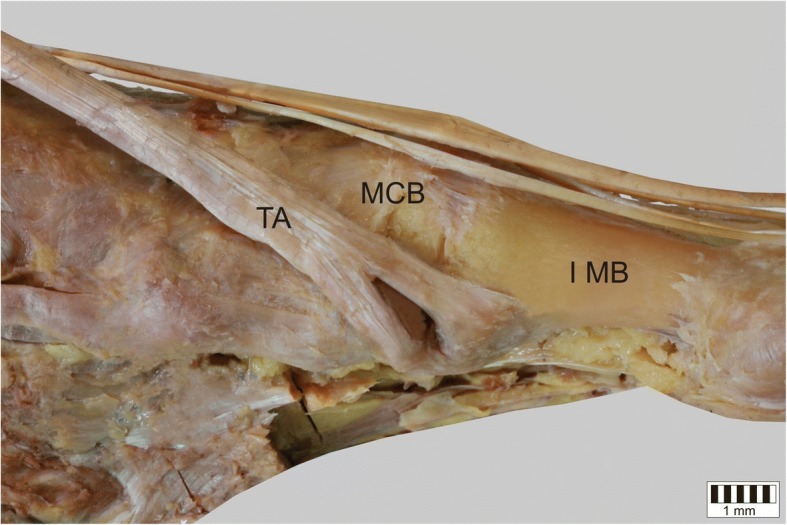
Type I tibialis anterior tendon. Medial view of the left leg. ***TA*** tibialis anterior tendon ***MCB*** medial cuneiform bone ***IMB*** first metatarsal bone

**Fig. 2:**
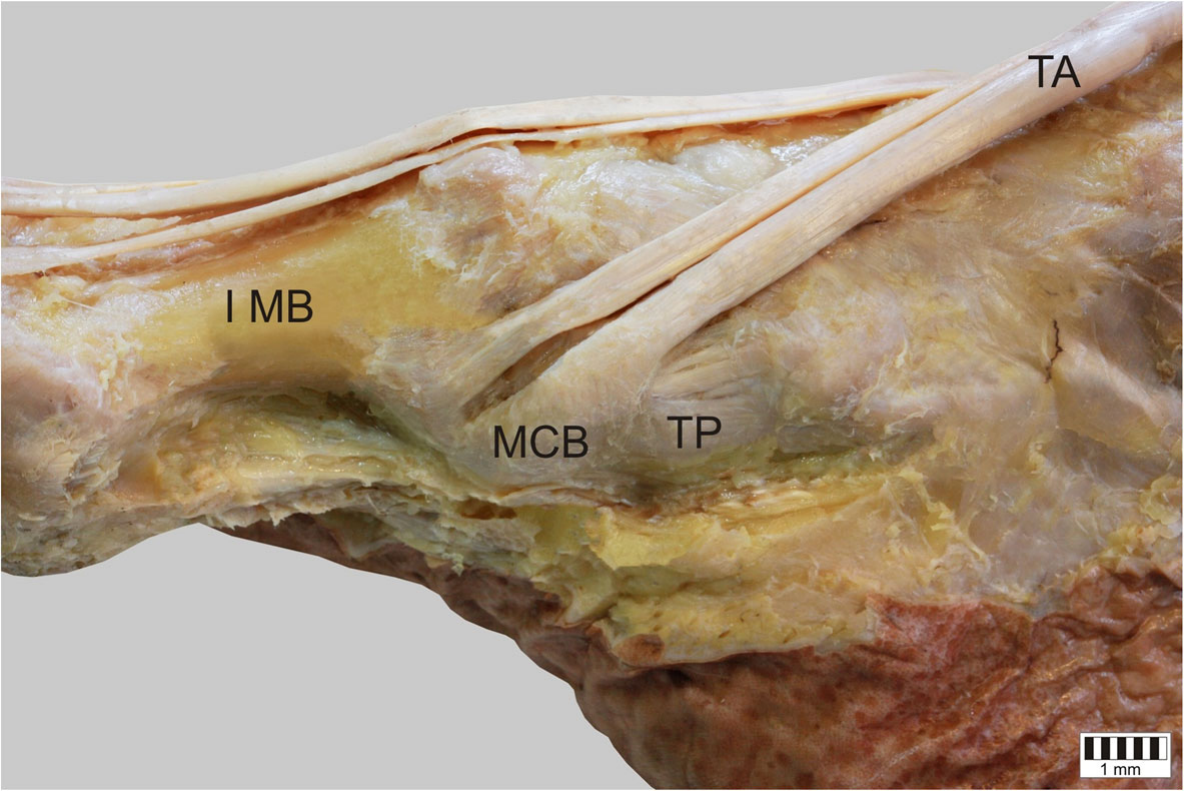
Type II tibialis anterior tendon. Medial view of the right leg. ***TA*** tibialis anterior tendon ***MCB*** medial cuneiform bone ***IMB*** first metatarsal bone ***TP*** tibialis posterior tendon

**Fig. 3:**
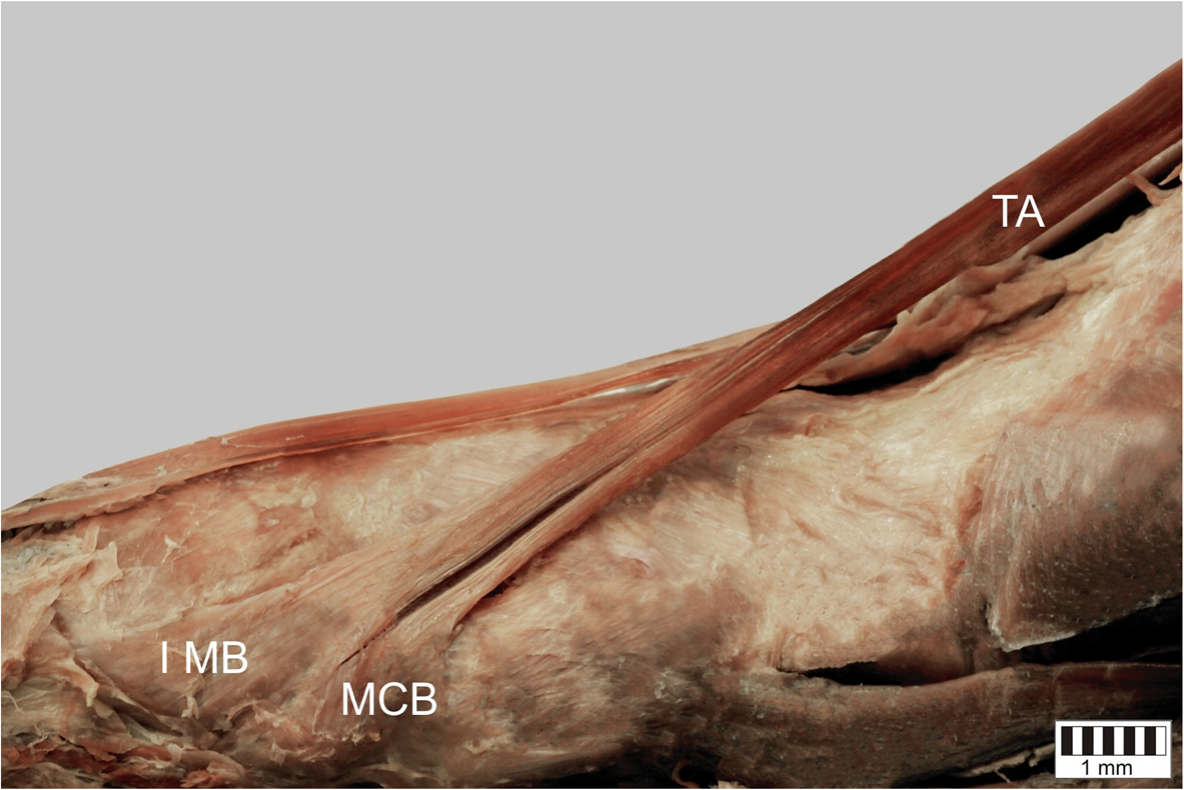
Type III tibialis anterior tendon. Medial view of the right leg. ***TA*** tibialis anterior tendon

**Fig. 4:**
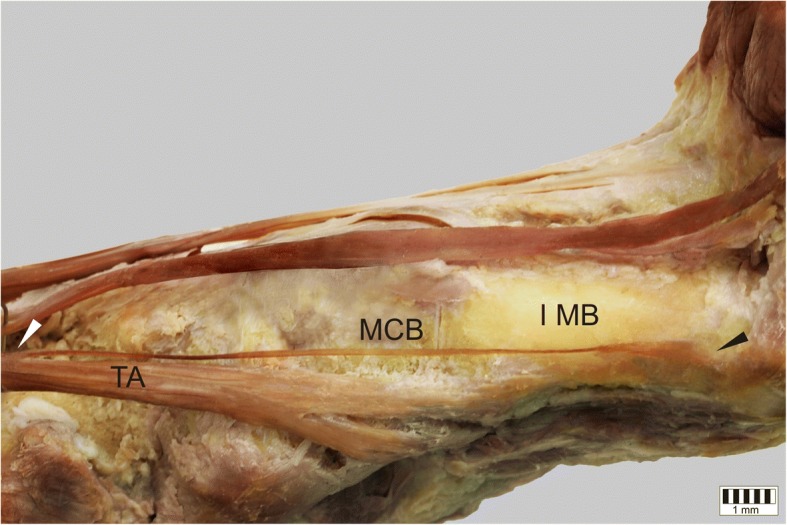
Type IV tibialis anterior tendon. Medial view of the left leg. ***TA*** tibialis anterior tendon ***MCB*** medial cuneiform bone ***IMB*** first metatarsal bone

**Fig. 5:**
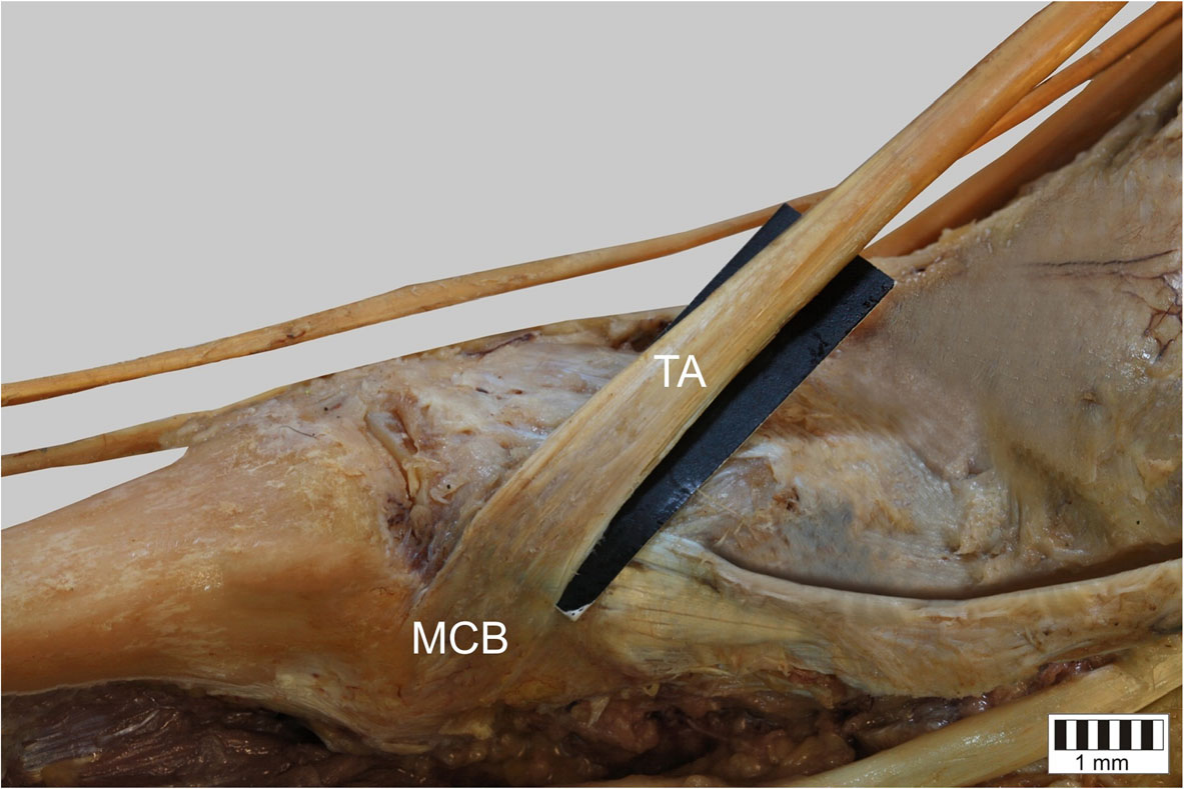
Type V tibialis anterior tendon. Medial view of the right leg. ***TA*** tibialis anterior tendon ***MCB*** medial cuneiform bone

The TA was present in all specimens. It could be classified into five types based on the morphology of the distal attachment:

- Type I – the tendon splits into two equal-size bands that insert to the medial cuneiform bone and base of the first metatarsal: observed in 31 cases – 31% (Fig. 1).
- Type II – the tendon splits into two bands that insert to the medial cuneiform bone (larger component) and the base of the first metatarsal (smaller component): observed in 24 cases – 24% (Fig. 2).
- Type III – the tendon splits into two bands that insert to the medial cuneiform bone (smaller component) and base of the first metatarsal (larger component): observed in 11 cases – 11% (Fig. 3).
- Type IV – the tendon trifurcates, inserting to the medial cuneiform bone (one band) and the first metatarsal (two bands - to the base and the shaft /distal part): observed in 2 cases – 2% (Fig. 4).
- Type V – a single band inserts to the medial cuneiform bone: observed in 32 cases – 32% (Fig. 5).

The morphometric parameters that differed significantly between types of the TAM are presented in Table 1.

**Table 1:** Differences in morphometric measurements between types of the TAT tendon. TAT [mean (SD)]

	Type I (*n* = 31) [mm]	Type II (*n* = 24) [mm]	Type III (*n* = 11) [mm]	Type IV (*n* = 2) [mm]	Type V (*n* = 32) [mm]	*p*-value
Distances to the origin of the first band	131.31 (27.59)^a^	129.94 (30.14)^a^	133.03 (31.41)	143.88 (27.92)	159.79 (38.48)^a^	0.0034
TA tendon first band width (insertion)	5.10 (1.97)^a,b^	7.38 (2.09)^a,b^	5.59 (1.46)^b^	8.11 (6.05)	10.65 (2.34)^b^	0.0000
TA tendon first band thickness (insertion)	2.39 (2.01)	2.17 (0.85)^a^	2.12 (0.95)	2.38 (0.16)	3.24 (1.09)^a^	0.0325

^a, b^ indicate groups that differ significantly between each other according to the post hoc test

### Sonographic study

**Fig. 6:**
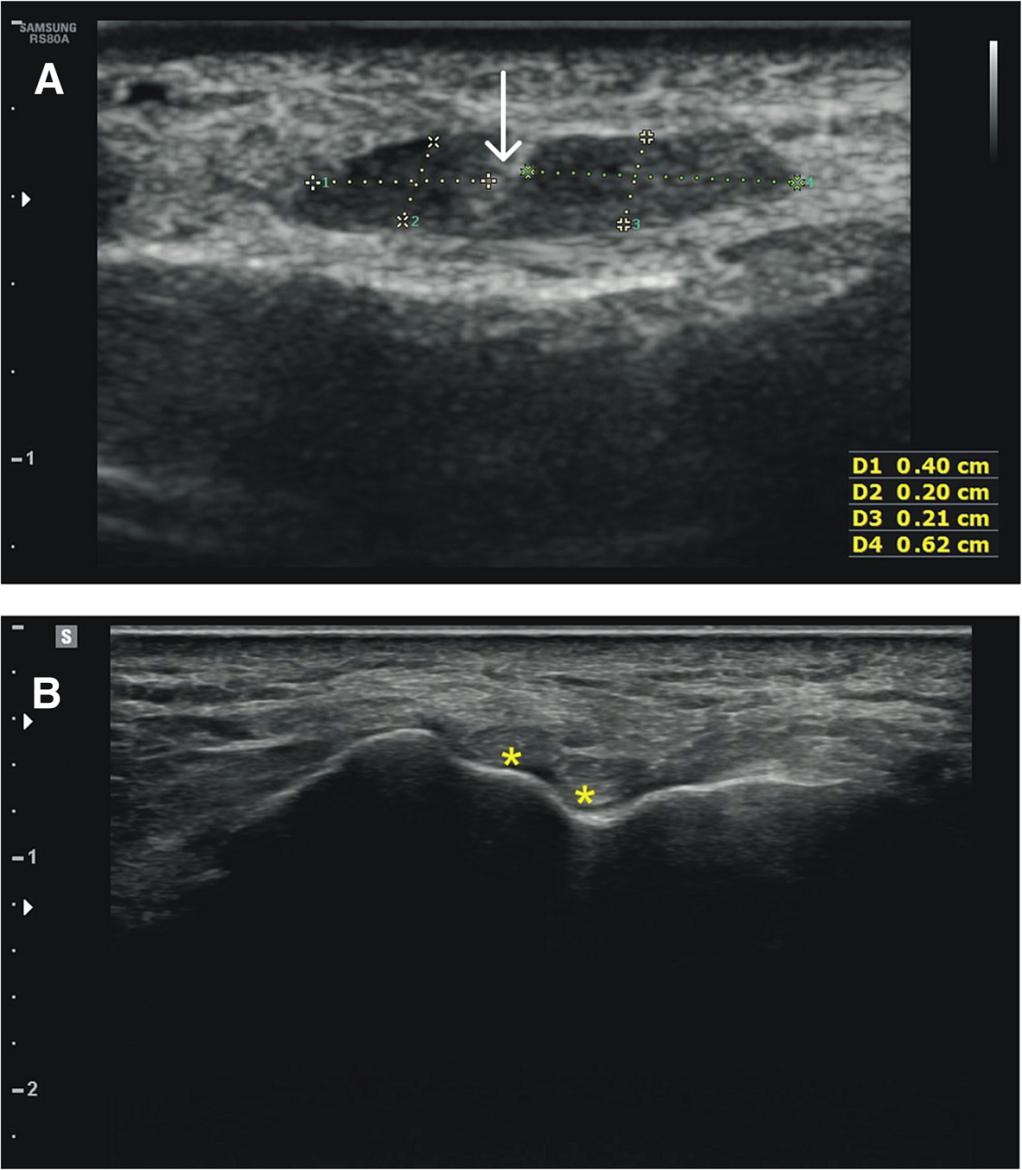
Type VI tibialis anterior tendon. Sonographic view of the cross-section of the tibialis anterior tendon in proximal section (**a**) and in distal section (**b**), close to the attachment. In proximal section (**a**) two components of the tendon are separated by a septa (arrow). In distal section (**b**) components (each marked with the asterix) are separated and travel in discrete notches on the medial cuneiform bone


In all volunteers, the TAM was recognised and presented no pathology. Based on the anatomical classification presented above, Type I was recognised in 20 limbs, Type II in 35, Type III in 13 and Type V in 20. Additionally, 12 lower limbs presented a type that was not seen in cadavers (Type VI): it was characterised by two equal-sized bands that both insert to the medial cuneiform bone (Fig. 6). In contrast, the Type IV observed in the cadavers was not found in the ultrasound study. Additionally, in all cases, the fibres of the TAT were found to rotate, with the superficial fibres winding to the medial side, and the deep fibres to the lateral side (film in Additional file 1).

**Additional file 1:** The rotation of the fibers of the TAT. (avi 3807 kb)


## Discussion

The most important asset of the present study is that it is the first to present a systematic classification of the TAT based on anatomical study which has also been partially confirmed by ultrasound examination. The resulting classification comprises a list of TAT types that not only differ visually, but they also display significant differences in their morphological characteristics. Such a systematisation can be used to improve the planning of surgical procedures and rehabilitation programmes.

Different types of insertions have been described in the literature [10, 12, 17–19]. The first classification of TAT insertions was introduced by Musiał [17] in 1963, who classified four types of insertions (Type 1–4). Type 1 was characterized by the tendon splitting into two equal-sized bands and then inserting to the medial cuneiform bone and the base of the first metarsal; this type was the second most frequently-observed type. The most common type of tendon, Type 2, splits into two bands that insert to the medial cuneiform bone (wider component) and the base of the first metatarsal (narrower component). The Type 3 tendon is characterised by a wide insertion onto the medial cuneiform and some rare fibers to the base of the first metatarsal, while the Type 4 tendon splits into two bands that insert to the medial cuneiform bone (narrower component) and base of the first metatarsal (wider component). In 1990, Arthornhurasook and Gaew Im [18] identified three types of insertion, two of which had been previously classified by Musiał. Brenner [19] classified five different insertions, and Willegger et al. [12] propose a modification of the classification introduced by Musiał [17]. The differences between our proposed classification and those given above are presented in Table 2.

**Table 2:** Comparison of studies

Type	Musiał (1963) [n/%]	Arhornhurasook and Gaew Im (1990) [n/%]	Brenner (2002) [n/%]	Willegger et al. (2017) [n/%]	Current study – anatomical part [n/%]	Current study – US part [n/%]
Two equal size bands that inserts to the MCB and FM	46/ 37.7	25/ 56.5	43/ 27.6	3/ 7.3	31/ 31	20/ 20
Wider component inserts to the MCB and narrower component inserts to the FM	69/ 56.5	12/ 27.3	71/ 45.5	20/ 48.8	24/ 24	35/ 35
Wider component inserts to the FM and narrower component inserts to the MCB	2/ 1.7	–	37/ 25.6	1/ 2.4	11/ 11	13/ 13
Wider component inserts to the FM and MCB and accessory slip to the distal part of the FM	5/ 4.1	–	–	–	2/ 2	–
Single band inserts to the MCB	–	7/ 15.9	2/ 1.3	–	32/ 32	20/ 20
Two bands inserts to the MCB	–	–	–	–	–	12/ 12
Single band inserts to the FM	–	–	3/ 1.9	–	–	–
Narrow inserts both MCB and FM	–	–	–	17/ 41.5	–	–

Accurate studies of the course of TAT and its insertion indicate that the previous classifications given in the literature do not encompass all the possible morphological variations of the TAT.

The most commonly-observed type in the anatomical part of the present study was Type V (32%), which was characterized by a single band inserted into the medial cuneiform bone. Additionally, it was the most distinct as far as the morphometric measurements are concerned because the tendon was significantly longer, thicker and wider. Interestingly, despite this type being so prevalent and morphologically distinct in the current classification (Table 1), it was described as rare by Arthronhursook and Gaew Im, as well as by Brenner [18, 19]. This type was also identified in 20% of the ultrasonography group.

The most common type observed in the ultrasonography group was Type II (35%), which was characterized by a wider component inserting into the medial cuneiform bone and a narrower component inserting to the base of the first metarsal. This type was also most frequently observed in anatomical studies by Brenner [19] and Willegger et al. [12]. Interestingly, Brenner introduced a classification of a type that had been not reported before, namely, one characterized by a single band inserting only to the first metatarsal bone. The radiological part of our research also revealed the existence of a potentially new type, which was categorized as Type VI. It comprised two equal size bands that both insert into the medial cuneiform bone (12%). The fibers of the TAT were found to medially rotate from the musculotendinous junction to their insertion on the medial cuneiform and first metarsal bone. This observation is consistent with those of Fennel and Philips [24].

Recent years have seen a significant increase in TAT ruptures. The patient usually suffers a minor trauma consisting of an excessive or forced unexpected plantar flexion which eccentrically stresses the contracting TAM [6]. The most common forms of TAT reconstruction are tendon transfer or allograft augmentation [6–9]. Our findings suggest that different types of insertion may result in the occurrence of slight differences in force distribution in the foot and ankle joints. Therefore, the biomechanics of the foot may be altered by the reconstructed joint being too tight or too loose. However, further biomechanical/clinical studies are required to confirm this, and our proposed classification may be of value in this work.

Our study has some limitations. Its main weakness is that the two study methods were not applied on the same samples, i.e. the sonographic and anatomical assessment of cadavers. It should be emphasised that although subjecting each cadaver to both assessment methods would be a more reliable method to confirm the practicality of ultrasound in evaluating TAT types, the aim of the present study was not to test the reliability of ultrasound evaluation per se, but to develop a detailed classification of the TAT that can be potentially applied in clinics. Nonetheless, this study helps raise awareness of “what and where” to look for, and offers a uniform classification and terminology to act as a foundation for communication with surgeons. Our new classification, including the addition of a new type (Type VI), can be of great value in determining the correct tendon reconstruction, and we believe that the adoption of such a new classification of the TAT would provide relevant anatomical knowledge which may help guide surgical procedures and rahabilitation process afterwards.

### Conclusion

The TAT insertion presents high morphological variability which can be observed in both cadavers and patients in vivo with the application of ultrasound. Our new classification systematizes this anatomical diversity and serves as a foundation for further biomechanical and clinical studies intended to improve the planning of surgical procedures and rehabilitation of patients after TAT injuries.

## Abbreviations

TAM: Tibialis anterior muscle
TAT: Tibialis anterior tendon
US: Ultrasonography
